# Recent Advances in Understanding the Control of Secretory Proteins by the Unfolded Protein Response in Plants

**DOI:** 10.3390/ijms14059396

**Published:** 2013-04-29

**Authors:** Shimpei Hayashi, Yuhya Wakasa, Fumio Takaiwa

**Affiliations:** Genetically Modified Organism Research Center, National Institute of Agrobiological Sciences, Kannondai 2-1-2, Tsukuba, Ibaraki 305-8602, Japan; E-Mails: hayapen@affrc.go.jp (S.H.); ywakasa@affrc.go.jp (Y.W.)

**Keywords:** secretory proteins, endoplasmic reticulum stress, unfolded protein response

## Abstract

The membrane transport system is built on the proper functioning of the endoplasmic reticulum (ER). The accumulation of unfolded proteins in the ER lumen (ER stress) disrupts ER homeostasis and disturbs the transport system. In response to ER stress, eukaryotic cells activate intracellular signaling (named the unfolded protein response, UPR), which contributes to the quality control of secretory proteins. On the other hand, the deleterious effects of UPR on plant health and growth characteristics have frequently been overlooked, due to limited information on this mechanism. However, recent studies have shed light on the molecular mechanism of plant UPR, and a number of its unique characteristics have been elucidated. This study briefly reviews the progress of understanding what is happening in plants under ER stress conditions.

## 1. Introduction

The endoplasmic reticulum (ER) is an important organelle that forms the basis of eukaryotic life phenomena. About 30% of nuclear-coded proteins which are synthesized on the rough ER mature in the ER lumen and are retained in the ER or transported though vesicular trafficking pathways [1]. The proper folding of such proteins is normally maintained at a relatively high level by folding machineries, including chaperones and modification enzymes in the lumen. The accumulation of unfolded proteins for various reasons disrupts ER homeostasis and has been referred to as “ER stress”. Eukaryotic cells have developed response systems called the “unfolded protein response (UPR)”, which alleviate ER stress and deal with its associated problems. To prove its importance, UPR has been associated with various human diseases, such as diabetes, inflammation and neurodegenerative disorders, and its molecular mechanisms have been investigated in detail in yeast and animals [2–4]. On the other hand, plant UPR research is in the early phase of development. Many phenomena in both basic and applied plant research have been associated with ER stress. In such cases, proteins can cause ER stress and unpredictably trigger UPR. Until recently, however, many plant researchers have not paid attention to the various effects caused by UPR, due to limited information being available on its molecular mechanism. Recent research has begun to reveal that plant UPR has serious effects on various cellular events and is involved in agronomically important traits, such as adaptability to environmental conditions and the productivity of useful materials [5–8]. Additionally, although the molecular mechanism of plant UPR is similar to those of yeast and animals in many respects, a number of plant-unique characteristics have also been revealed. As practical or general knowledge, understanding what happens in plants under ER stress conditions is of importance. Plant UPR research has been mainly conducted in Arabidopsis, tobacco and rice. In particular, a part of the research performed on rice began to solve practical problems accompanied with the application of a membrane transport system in rice seeds, and consistent and systematic knowledge has consequently begun to be acquired. This study has introduced topics that are focused on rice.

## 2. Occurrence of ER Stress

In plants, ER stress is induced by treatments that disrupt protein folding, such as heat shock, reducing agents (e.g., dithiothreitol), amino acid analogs and inhibitors of the glycosylation enzyme (e.g., tunicamycin). These treatments also induce ER stress in yeast and animals, suggesting that the inductive mechanism of ER stress is essentially the same in eukaryotes. In addition to these treatments, the expression of transgenes may cause ER stress.

**Figure 1:**
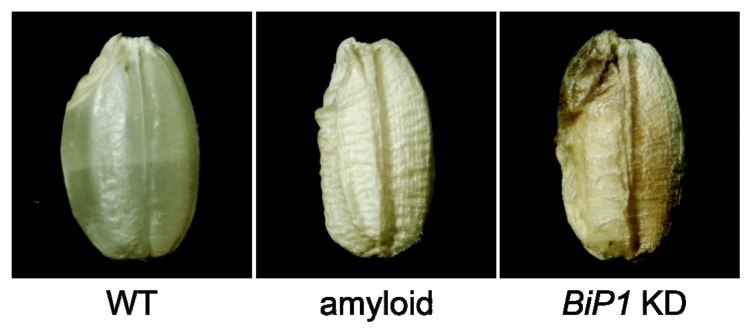
Endoplasmic reticulum (ER) stress-related phenotypes of transgenic rice seeds. Transgenic rice seeds in which a mammalian amyloid is produced as an ER retention protein or the *BiP1* gene is knocked down (KD) specifically in the endosperm. These transgenic rice seeds show the following phenotypes: opaque and shrunken phenotype, decreased starch content, decreased seed storage proteins (protein and mRNA levels) and induction of ERQC-related genes.

One of the main characteristics of the plant ER is the use of the lumen for production. Certain types of seed storage proteins synthesized as secretory proteins are retained in the ER lumen and accumulate as protein bodies [9]. It is assumed that vigorous protein secretion is associated with a risk of unfolded protein generation. However, despite the large amount of proteins, storage events normally do not induce detectable levels of UPR. This suggests a highly sophisticated mechanism to control the quality of secretory proteins in plants. Using the protein storage pathway, our research group succeeded in accumulating high amounts of transgene-products in the protein bodies of the rice endosperm [10,11]. However, in some cases, such transgene-products may cause unfavorable effects on rice seeds, as shown in Figure 1 [12–14]. Abnormal seeds with floury and shrunken features showed the strong induction of ER stress-responsive gene expression, suggesting that the accumulation of unfolded proteins is responsible for such phenotypes. Additionally, rice seeds in which the main ER chaperon protein BiP1 was knocked down specifically in the endosperm showed some similar features [13], which suggests that the protein storage events in wild-type rice seeds are built on by the aid of various chaperone proteins and folding enzymes, including BiP proteins. Some ER chaperones of Arabidopsis also contributed to the efficient maturation and secretion of proteins during disease responses [15–17]. Thus, the induction of ER stress is dependent on the physicochemical properties and expression levels of secretory proteins. However, clear rules on the inducibility of ER stress by transgene-products have not been established. Consistent with the sensitivities of plants to dithiothreitol and tunicamycin, the characterization of other ER-stressed transgenic rice seeds suggests that the involvement of disulfide bonds or glycosylation in the production of transgenes is important parameters [18,19]. Other examples of detrimental effects associated with ER stress include cell death. Treatment of plants with tunicamycin leads to loss of cell viability accompanied by typical hallmarks of programmed cell death [20].

An important unresolved issue is whether the phenotypes observed in ER stressed plants are due to the effects of UPR or disordered ER functions. It has been assumed that UPR reduces the damage accompanying ER stress and simultaneously has unfavorable side effects. The constitutive activation of UPR signaling components was shown to cause detrimental effects on the growth of rice plants without ER stress treatments [21,22]. To clarify this issue, UPR signaling pathways need to be understood at the molecular level.

## 3. Signaling Components

The accumulation of unfolded proteins in the ER lumen is sensed by ER membrane-localized sensor proteins and, consequently, the signal is transduced to the nucleus. This signal is also assumed to induce cellular events without *de novo* transcription. Signaling components in plants have been identified with reference to those in yeast and animals. Some of these components are conserved between plants and animals. However, a number of plant-unique characteristics have been found.

### 3.1. ER Stress Sensors

Several types of ER stress sensors have been identified in eukaryotes [23]. ER localized transmembrane protein, IRE1, is the most conserved ER stress sensor in eukaryotes and is the only one in yeast. Besides IRE1, transmembrane proteins PERK and ATF6 have also been identified as ER stress sensors in animals. The orthologs of IRE1 and ATF6 have been identified in plants, whereas the plant counterpart of PERK has not yet been found by a sequence search [24–26].

#### 3.1.1. IRE1

**Figure 2:**
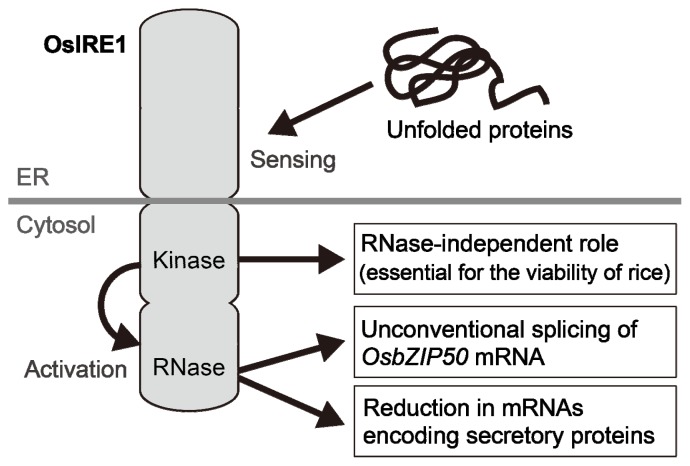
Multiple roles of IRE1 in rice. The kinase activity of IRE1 is assumed to contribute to activation of the RNase domain. RNase activity is involved in the unconventional mRNA splicing of OsbZIP50 and the reduction of mRNAs encoding secretory proteins, such as PR proteins. Kinase activity plays some role independent of RNase activity that is essential for the viability of rice.

The *N*-terminal portion of IRE1 resides in the ER lumen, and the *C*-terminal portion resides in the cytosol [23] (Figure 2). The cytosolic region contains a serine/threonine kinase domain and an endo-ribonuclease (RNase) domain. The accumulation of unfolded proteins in the ER lumen leads to the clustering of IRE1, autophosphorylation of the kinase domain and consequent activation of RNase. This RNase activity mediates the unconventional splicing of the mRNA encoding the key transcription factors, HAC1 (yeast), XBP1 (animals), AtbZIP60 (Arabidopsis) or OsbZIP50 (rice), and the spliced forms of these mRNAs are translated as active forms [27–31]. In animals, IRE1 plays multiple roles other than the cleavage of *XBP1* mRNA and serves as a branch point for UPR signaling [32]. Genetic analysis of transgenic rice plants, in which the genomic *IRE1* gene has been edited by homologous recombination, recently demonstrated that the kinase activity of IRE1 played a vital role independent of RNase activity [33]. This finding suggests that plant IRE1 interacts with some signaling components in a kinase activity-dependent manner and mediates multiple signaling pathways.

#### 3.1.2. ATF6-Like Transcription Factors

In animals, ER stress is also sensed by the transcription factor, ATF6, a transmembrane protein activated by ER stress-mediated proteolysis via site 1 and 2 proteases in the Golgi apparatus [34,35]. In plants, AtbZIP17 and AtbZIP28 in Arabidopsis and OsbZIP39 and OsbZIP60 in rice are the counterparts of ATF6 [22,26,31,36]. The truncated forms of these plant transcription factors induce some ER stress-responsive genes without ER stress treatments. The currently known molecular aspects of these types of transcription factors seem to be similar between animals and plants.

### 3.2. Transcription Factors Regulated by IRE1-Mediated Unconventional mRNA Splicing

IRE1 activation by ER stress mediates the unconventional splicing of mRNAs encoding transcription factors, leading to a transition to their active forms as a result of frame shifting. IRE1-mediated mRNA splicing is required to generate the transcriptional activation domains of yeast HAC1 and animal XBP1 [27,28]. In contrast to HAC1 and XBP1, the activation domains of plant counterparts are located within a region that is not affected by IRE1-mediated splicing [30,31]. The unspliced form of Arabidopsis AtbZIP60 and rice OsbZIP50 proteins cannot be translocated to the nucleus, whereas the spliced forms efficiently localized in the nucleus. These observations suggest that IRE1-mediated splicing allows these plant transcription factors to be localized in the nucleus. It has been experimentally demonstrated in rice that the alternative *C*-terminal domain of the spliced form is required for efficient nuclear localization [31]. Although the structures of mRNA processed by IRE1 are similar, the effects of splicing on their translation products are very different. This interspecies diversity reflects flexibility in the molecular evolution of IRE1-mediated signaling.

In contrast to ATF6-like transcription factors, the activation of OsbZIP50 and AtbZIP60 requires *de novo* protein synthesis. Therefore, this activation is predicted to respond more slowly than that of ATF6-like transcription factors. Additionally, OsbZIP50 and AtbZIP60 were shown to form heterodimers with ATF6-like transcription factors [21,37].

### 3.3. *cis*-Elements

As mentioned above, plants and animals exhibit similarities between their UPR-related transcription factors. Additionally, the UPR-related *cis*-elements identified in animals have also been found in the promoter regions of plant ER stress-responsive genes [38]. Similar to ATF6, the Arabidopsis AtbZIP28 directly interacts with the CACG-box of ERSE (CCAAT-N10-CACG) with assistance from the NF-Y transcription factor [37]. Additionally, mUPRE (TGACGTGR) is a representative element that binds to XBP1 [39]. Recently, the *cis*-element that directly interacts with the plant counterpart of XBP1 was identified in rice using an unbiased approach involving chromatin immunoprecipitation and the electrophoretic mobility shift assay [21]. The identified *cis*-element pUPRE-II was shown to bind to OsbZIP50 and markedly contribute to ER stress-induced gene expression. The sequence of pUPRE-II was partially similar, but not identical to those of mUPRE. Additionally, unlike mUPRE, pUPRE-II also strongly bound to the rice counterpart of ATF6, OsbZIP60, and mediated OsbZIP60-induced gene expression. These findings strongly suggest that the transcriptional regulation system of ER stress-responsive genes in plants has evolved in a unique way. Although OsbZIP50 and OsbZIP60 partially share *cis*-elements, the requirements for transcriptional activation between them are different. To explain the detailed molecular mechanism of ER stress-induced gene expression, identifying the unknown factors that control these transcription factors is required. Although several other UPR-related *cis*-elements have been proposed [40,41], interactions between these elements and transcription factors have not been adequately evaluated. Some may also contribute to transcriptional regulation.

## 4. Upregulation of Gene Expression

ER stress-induced genes have been identified by DNA microarray analyses in Arabidopsis and rice [12,13,22,30,36,42]. Analyses of mutant or genetically modified plants demonstrated that many of these genes are induced by UPR mediated by the signaling components described above. Plant UPR-related transcription factors mainly induce the gene expression of ER quality control (ERQC)-related factors, such as BiP, which facilitate protein folding in the ER lumen. Consistent with the partial sharing of *cis*-elements, the target genes of OsbZIP50, OsbZIP39 and OsbZIP60 were shown to be partially overlapped [21,22]. However, these transcription factors also have specific targets, suggesting the division of roles between them. Beside ERQC-related genes, various other genes are induced by plant UPR. They include some transcription factors, which suggests the existence of transcriptional cascades triggered by ER stress. Additionally, some of the genes upregulated by UPR have also been identified as components in other signaling pathways. The involvement of AtbZIP17 and AtbZIP28 has been reported in Arabidopsis brassinosteroid signaling [36]. In the case of rice, *OsWRKY45*, which is a key transcription factor mediating the salicylic acid (SA) response, and *OsbZIP8*, which may be involved in the abscisic acid response, were shown to be induced by UPR [12,22,43,44].

**Figure 3:**
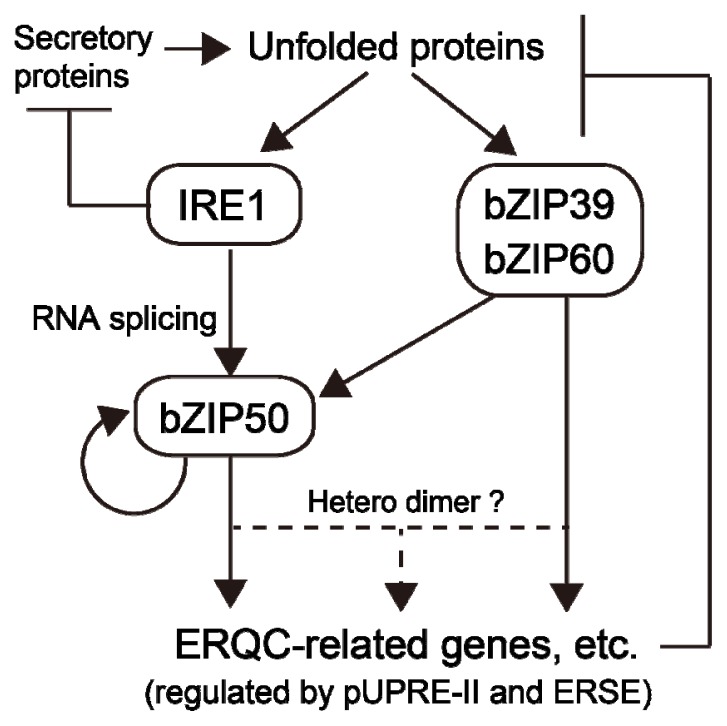
Model of the relationship between ER stress sensors and OsbZIP50. A large amount of secretory proteins increases unfolded proteins in the ER lumen. The accumulation of unfolded proteins induces active forms of the ER stress sensors OsIRE1, OsbZIP39 and OsbZIP60. Proteolytically activated OsbZIP39 and OsbZIP60 induce the expression of ER quality control (ERQC)-related genes and *OsbZIP50*. OsbZIP50, activated by OsIRE1-mediated mRNA splicing, induces its own expression and the expression of ERQC-related genes. Some OsbZIP50 and OsbZIP39 or OsbZIP60 form heterodimers. The inducibility of gene expression by these transcription factors is regulated by *cis*-elements, such as pUPRE-II and ERSE. ERQC-related gene products contribute to the reduction in unfolded proteins and the end of strong UPR. In addition to the activation of *OsbZIP50* mRNA, OsIRE1 suppresses a certain type of secretory protein and, thereby, avoids worsening ER stress.

The gene expressions of *OsbZIP50* and *AtbZIP60* are also induced by ER stress and are assumed to be partially controlled by ATF6-like transcription factors [31]. Additionally, OsbZIP50 activated by IRE1-mediated mRNA splicing induces its own transcription and, thereby, amplifies the ER stress signal [31]. These findings demonstrate that the activation of OsbZIP50 is strictly regulated by multiple ER stress sensors transcriptionally and post-transcriptionally (Figure 3). Since the long-term activation of OsbZIP50 caused growth defects in plants [21], this regulation system, which is suited to cease signal transduction as soon as the stress is relieved, is very rational.

The accumulation of transcripts upregulated by UPR becomes indicators of ER stress and UPR. For instance, the induction of the *BiP4* (*Os05g0428600*) or *SAR1*-like gene (*Os06g0225000*) serves as a good indicator of UPR activation in rice, because their induction is highly specific to UPR, and their promoter regions have been relatively well characterized [14,21,31]. In the case of Arabidopsis, the induction of *BiP3* (*At1g09080*) may be suitable as the indicator [30]. In addition to these genes, the accumulation of the spliced forms of *OsbZIP50* or *AtbZIP60* mRNA can be used to detect ER stress. The orthologs of these genes could be used for the same purposes in many of the other plant species.

## 5. Downregulation of Gene Expression

Plant UPR also downregulates the expression levels of various genes. Microarray analyses with rice plants showed that many of the genes downregulated by UPR encode pathogenesis-related (PR) proteins, which are assumed to confer antibiotic properties to plants and that a reduction in their mRNA levels was largely dependent on IRE1 [43]. A further study demonstrated that such downregulation of PR gene expression was not dependent on OsbZIP50 [33]. Furthermore, transgenic rice plants in which the RNase activity of IRE1 is selectively eliminated by homologous recombination showed a moderate reduction in PR gene mRNAs [33]. These results indicate that the reduction is at least partially dependent on IRE1 RNase activity in an OsbZIP50-independent manner. IRE1 has been proposed to participate in the degradation of various mRNAs in animals, and this system is called the “regulated IRE1-dependent decay of mRNAs (RIDD)” [45]. Many of the degraded mRNAs encode secretory proteins that pass through the ER lumen. Similar to this, the PR genes downregulated by ER stress in rice have also been predicted to encode secretory proteins. Although it remains undetermined whether the mRNAs of these rice PR genes are reduced by degradation, these findings imply that this is RIDD in plants. Recently, Mishiba *et al.* reported that mRNAs of many PR genes in Arabidopsis were also downregulated in an IRE1-dependent manner and that these are post-transcriptional events, demonstrating for the first time the existence of RIDD in plants [46]. Additionally, the report statistically demonstrated that most of these downregulated genes are predicted to encode secretory proteins. The RIDD system presumably contributes to avoiding worsening ER stress by reducing the loading of secretory proteins into the ER lumen. In animals, the ER stress sensor, PERK, serves as a suppressor of translational events in response to ER stress. Although the counterparts of PERK have not been found in plants, plants also have a system to control the amount of proteins at the pre-translational level.

In addition to RIDD, post-translational regulation systems, such as the proteasome-mediated proteolysis of ER-localized proteins, called “ER-associated degradation”, and autophagy, have also been shown to be involved in the quality control of proteins in the ER [47,48]. The mRNA and protein levels of some seed storage proteins in rice, which are also secretory proteins, were significantly reduced under severe ER stress conditions [12,13]. The expression level of the seed storage proteins may also be controlled by RIDD and/or these post-translational regulation systems.

## 6. Interaction with Other Life Phenomena

Conditions within the ER influence most secretory proteins that pass through it. Therefore, eukaryotic cells must strike a balance between UPR, which changes the conditions in the ER, and other considerations associated with protein secretion. Interferences between UPR and other plant responses have been reported.

**Figure 4:**
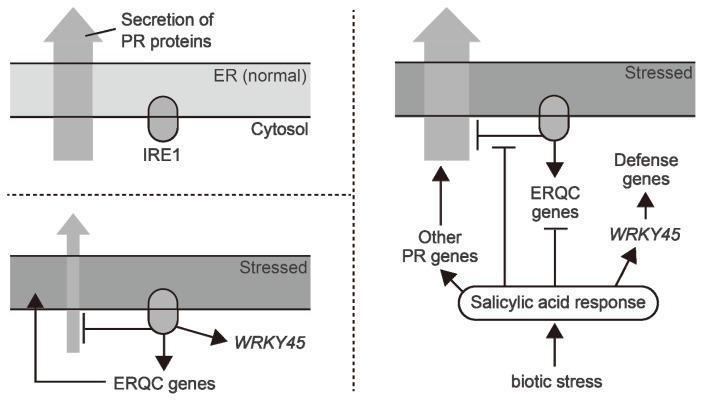
Model of the relationship between IRE1-mediated unfolded protein response (UPR) and the defense system in rice. Under normal conditions, certain types of PR proteins are secreted through the ER and maintain basic disease resistance (upper left panel). When ER stress conditions arise, to relieve this stress, IRE1-mediated UPR pathways induce ERQC-related factors and reduce the gene expression of PR proteins (lower left panel). Additionally, the IRE1-mediated pathway induces the gene expression of OsWRKY45, which improves disease resistance. When the salicylic acid (SA) response is activated concomitantly with UPR, most of the aforementioned effects of IRE1-mediated UPR are suppressed (right panel). The SA response induces other types of PR genes and the *OsWRKY45* gene in an IRE1-independent manner and activates the accumulated OsWRKY45 to further improve disease resistance.

An important topic is that of the relationship between UPR and the defense system of plants. As described above, UPR induces the expression of ERQC-related genes and reduces the mRNA levels of some PR genes. Interestingly, activation of the SA response, which plays a central role in plant defense, was shown to suppress the induction of ERQC-related genes and the reduction in PR gene expression by UPR [43] (Figure 4). In such a case, *OsWRKY45* transcripts induced by UPR are not reduced by the SA response, because *OsWRKY45* is also induced by SA independently of UPR. Resistance to some diseases is improved by the overexpression of *OsWRKY45* [49]. Therefore, the OsWRKY45 protein, which accumulated in response to ER stress, may offset the risk associated with the reduction in PR proteins. IRE1 is involved not only in the expression of ERQC-related genes, but also in the expression of defense-related genes (OsWRKY45 and PR genes). Additionally, the different manner of involvement of IRE1 in the defense system was also reported in Arabidopsis and tobacco [50,51]. In addition to defense, a HSP70-encoding gene induced via the IRE1-OsbZIP50 pathway is critically involved in the hybrid sterility between two rice subspecies [52]. IRE1 is encoded by a single gene in rice, whereas Arabidopsis has two IRE1 paralogues. Interestingly, rice plants in which *IRE1* is severely knocked down showed lethality, whereas a double disruption mutant of the Arabidopsis *IRE1* paralogues did not [30,33]. These findings suggest that, over the course of evolution, IRE1 has been incorporated into species-specific systems and interferes in a variety of life phenomena.

## 7. Conclusions

There are a number of examples where the expression of transgenes or some experimental circumstance has caused unexpected side effects due to ER stress. To avoid misreading experimental data or to find new insights, we should always consider the potential effects of ER stress. Additionally, detailed understanding of plant ER stress will contribute to the development of agricultural or industrial use of plants as production platforms for recombinant proteins by reducing negative effects associated with ER stress and by promoting positive effects of UPR.
